# Optimising brain age estimation through transfer learning: A suite of pre‐trained foundation models for improved performance and generalisability in a clinical setting

**DOI:** 10.1002/hbm.26625

**Published:** 2024-03-04

**Authors:** David A. Wood, Matthew Townend, Emily Guilhem, Sina Kafiabadi, Ahmed Hammam, Yiran Wei, Ayisha Al Busaidi, Asif Mazumder, Peter Sasieni, Gareth J. Barker, Sebastien Ourselin, James H. Cole, Thomas C. Booth

**Affiliations:** ^1^ School of Biomedical Engineering and Imaging Sciences, Rayne Institute King's College London London UK; ^2^ King's College Hospital NHS Foundation Trust London UK; ^3^ Guy's and St Thomas' NHS Foundation Trust London UK; ^4^ Department of Neuroimaging, Institute of Psychiatry, Psychology, and Neuroscience King's College London London UK; ^5^ Dementia Research Centre, Institute of Neurology University College London London UK; ^6^ Centre for Medical Image Computing, Department of Computer Science University College London London UK

**Keywords:** brain age, deep learning, foundation model, MRI, transfer learning

## Abstract

Estimated age from brain MRI data has emerged as a promising biomarker of neurological health. However, the absence of large, diverse, and clinically representative training datasets, along with the complexity of managing heterogeneous MRI data, presents significant barriers to the development of accurate and generalisable models appropriate for clinical use. Here, we present a deep learning framework trained on routine clinical data (*N* up to 18,890, age range 18–96 years). We trained five separate models for accurate brain age prediction (all with mean absolute error ≤4.0 years, *R* ^2^ ≥ .86) across five different MRI sequences (T_2_‐weighted, T_2_‐FLAIR, T_1_‐weighted, diffusion‐weighted, and gradient‐recalled echo T_2_*‐weighted). Our trained models offer dual functionality. First, they have the potential to be directly employed on clinical data. Second, they can be used as foundation models for further refinement to accommodate a range of other MRI sequences (and therefore a range of clinical scenarios which employ such sequences). This adaptation process, enabled by transfer learning, proved effective in our study across a range of MRI sequences and scan orientations, including those which differed considerably from the original training datasets. Crucially, our findings suggest that this approach remains viable even with limited data availability (as low as *N* = 25 for fine‐tuning), thus broadening the application of brain age estimation to more diverse clinical contexts and patient populations. By making these models publicly available, we aim to provide the scientific community with a versatile toolkit, promoting further research in brain age prediction and related areas.

## Graphical abstract

Leveraging transfer learning, we introduce a deep learning framework trained on vast clinical MRI data to predict brain age with high accuracy across five MRI sequences. Our versatile, open‐source models, effective even with limited data, promote wider application in diverse clinical scenarios and advance brain age research.

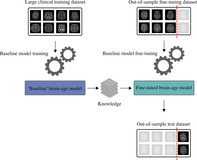

## 1 INTRODUCTION

Brain age estimation uses neuroimaging data to determine an individual's biological age and has shown potential as a biomarker of neurological health (Cole & Franke, 2017). The underlying assumption is that typical brain development and ageing processes follow predictable trajectories and that divergences from these patterns can signal neurodegenerative processes or accentuate age‐related brain health issues. Such deviations are quantified in individuals by comparing their estimated brain age with their chronological age, resulting in a brain‐predicted age difference (brain‐PAD) (Cole et al., 2017; Smith et al., 2019). A positive brain‐PAD, indicating an older‐appearing brain compared to actual chronological age, has been associated with numerous neurological and psychiatric conditions (Franke & Gaser, 2019) and future health outcomes (Biondo et al., 2022; Elliot et al., 2021; Popescu et al., 2020). These findings underscore the possible value of brain age estimation as a non‐invasive tool for early diagnosis, patient stratification, and monitoring of disease progression.

A key goal of brain age research is to ultimately benefit patients (Kelly et al., 2019). However, realising this goal will involve overcoming several challenges. One challenge is the lack of representativeness of research datasets (Agarwal et al., 2023; Agarwal & Wood et al., 2023; Din et al., 2023), particularly public datasets commonly used for training brain age models. This applies not only to the demographics of the study participants, but also to the nature of the MRI data (e.g. sequences and acquisition parameters) and to the data quality. Another related challenge is training sample size, whereby smaller samples are less likely to be representative of downstream test sets, hence limiting generalisability. One option to overcome this would be to train models from scratch using local data that are more representative of the target population. However, this is not possible in many circumstances, where local data suitable for training are not routinely acquired, budgets for large‐scale data collection are limited, or diseases have a low prevalence.

A promising avenue for making machine learning models more representative and generalisable is transfer learning. Put simply, the idea is to transfer what is learned from one machine learning task to another task (Chelliah et al., 2024; Zhuang et al., 2020). This type of ‘domain adaptation’ typically involves ‘fine‐tuning’ the original model using a subset of labelled data from the second task. Using a pre‐trained model in this way aims to benefit downstream model training speed (i.e. the time to convergence) and performance compared to training a new model from scratch, where the network weights and biases are initialised randomly. Transfer learning is an established technique in natural language processing (Devlin et al., 2018; Howard & Ruder, 2018) and computer vision (Yosinski et al., 2014) and is becoming increasingly popular in neuroimaging (Agarwal et al., 2021; Ardalan & Subbian, 2022). Transfer learning has already been applied with some success in the context of brain age (Chen et al., 2020; Jonsson et al., 2019; Leonardsen et al., 2022), showing how using pre‐trained models can improve downstream prediction in, for example, specific disease groups. However, these studies all used research cohorts with high‐quality MRI and only focused on a single modality (T_1_‐weighted or diffusion‐weighted).

Here, we aimed to use transfer learning to overcome some of the limitations of previous brain age studies, building on our previous work showing how convolutional neural network (CNN) models of brain age can be trained to predict age from various clinical‐grade (i.e. non‐volumetric) MRI modalities (Wood et al., 2022a). We trained, at scale, different brain age models for different modalities, using data from a large and clinically representative dataset, with the goal of generating a framework for transferring knowledge (i.e. pre‐trained models) to a breadth of possible scenarios.

We hypothesised that we could train accurate age prediction ‘baseline’ models from clinical‐grade scans of five different MRI sequences (T_2_‐weighted, T_2_‐FLAIR, T_1_‐weighted, diffusion‐weighted (DWI), and gradient‐recalled echo (GRE) T_2_*‐weighted) and that the most accurate performance could be achieved by combining predictions with an ensemble of all five models. We further hypothesised that transfer learning could be used to improve generalisability in a variety of downstream scenarios, namely out‐of‐sample testing on (i) data with equivalent acquisition parameters acquired at a different site, (ii) data with the same modality but a different primary acquisition plane, or (iii) data of a different modality from the baseline pre‐trained model. This was done by comparing prediction performance of baseline models with no fine‐tuning versus fine‐tuned models or when training on the new data from scratch (i.e. no transfer learning). Finally, we explored the necessary sample sizes required to achieve improved performance during fine‐tuning.

## 2 MATERIALS AND METHODS

### 2.1 Datasets

All data were de‐identified. The UK National Health Research Authority and Research Ethics Committee approved this retrospective study (IRAS ID 235,658, REC ID 18/YH/0458).

#### 2.1.1 Head MRI clinical datasets for brain age model development

The dataset used in this study was the same as that used in previous brain age modelling work (Wood et al., 2022a). Briefly, all 81,936 adult (≥18 years old) head MRI examinations performed in the UK at Guy's and St Thomas' NHS Foundation Trust (GSTT) and King's College Hospital NHS Foundation Trust (KCH) between 2008 and 2019 were collected retrospectively. The MRI scans were performed using Ingenia 1.5 T (Philips Healthcare, Eindhoven, Netherlands), Aera 1.5 T (Siemens, Erlangen, Germany), Signa 1.5 T HDX (General Electric Healthcare, Chicago, USA), or Skyra 3 T (Siemens, Erlangen, Germany) scanners. The corresponding free‐text radiology reports produced by 17 expert neuroradiologists were extracted from the Computerised Radiology Information System (CRIS) (Healthcare Software Systems, Mansfield, UK). These reports were predominantly unstructured, typically consisting of 5–10 sentences describing the image interpretation, along with comments regarding the patient's clinical history and recommended actions for the referring physician.

The number of MRI sequences acquired during each examination in this dataset ranged from 1 to 8 (Figure A1 in Appendix A). The most frequently acquired sequence and orientation combinations were axial T_2_‐weighted, axial DWI, coronal T_2_‐FLAIR, sagittal T_1_‐weighted, and axial GRE T_2_*‐weighted images, performed in 97.2%, 78.5%, 66.1%, 43.8%, and 43.7% of examinations, respectively. We elected to develop individual ‘baseline’ brain age models for these five common sequences and explore transfer learning using public datasets (IXI, OASIS‐3, ADNI—described in detail below) to facilitate brain age modelling for scans that appeared with lower frequency in our study dataset (e.g. susceptibility‐weighted and proton density‐weighted images). Our baseline brain age models therefore serve a dual purpose. First, they can be directly used to predict brain age using the specific MRI sequence they were trained on. Second, they serve as foundation models for transfer learning, allowing further tuning on new, possibly smaller datasets, to improve generalisability performance or to adapt to a range of other MRI sequences not seen during the initial training.

A subset of ‘radiologically normal for age’ examinations was identified using a dedicated transformer‐based neuroradiology report classifier (Wood et al., 2020, 2021; Wood et al., 2022b). This model was trained using a large dataset of neuroradiology reports from KCH (*N* = 5000) which had been annotated by a team of five expert neuroradiologists (UK consultant grade; US attending equivalent) as either ‘radiologically normal for age’ or ‘radiologically abnormal for age’ based on well‐defined criteria (Benger et al., 2023; Wood et al., 2020; Wood et al., 2022b). Briefly, findings that could lead to a subsequent clinical intervention were labelled as ‘abnormal’ (a referral for case discussion at a multidisciplinary team meeting was considered the minimal intervention). Importantly, the abnormal category included findings deemed ‘excessive for age’ (e.g. excessive volume loss and extensive small vessel disease observed on T_2_‐weighted images). In this previous work, the classifier demonstrated near‐perfect accuracy (area under the receiver operating characteristic curve [AUC] = 0.991) on a testing dataset of 500 radiology reports from KCH and generalised to an external testing dataset of 500 reports from GSTT (AUC = 0.990).

**FIGURE 1:**
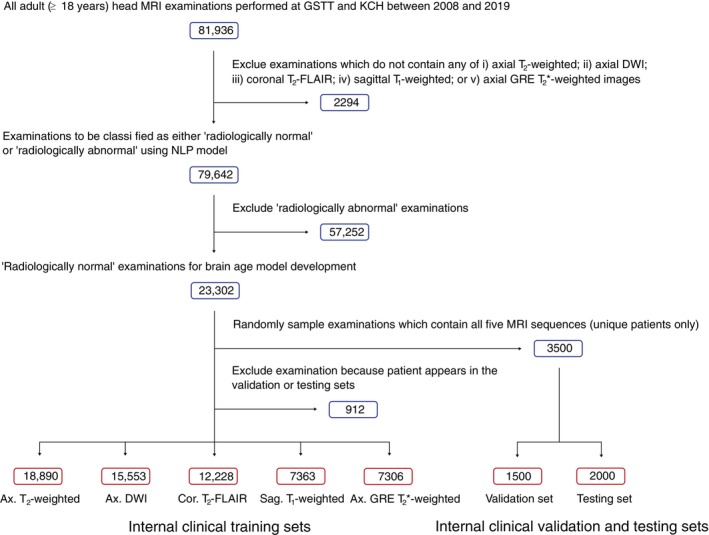
Flow chart depicting the process of creating the internal clinical datasets used in this study. To capture the diversity of examinations seen in clinical practice, we did not exclude any reported examinations on the basis of image quality.

In the current study, a total of 22,302 examinations from the larger dataset were identified as ‘radiologically normal for age’ and included for baseline brain age model development (male/female = 9299/14,003, mean age = 43.6 ± 15.3 years, age range = 18–96 years). Validation (*N* = 1500) and testing (*N* = 2000) datasets were created by randomly selecting 3500 examinations (*N* unique patients = 3500) from the subset that included all five MRI sequences (Figure 1). We removed any overlapping instances of patients that were present in the testing or validation datasets from the remaining pool of examinations, resulting in a training dataset of 18,890 examinations comprising different numbers of each type of MRI sequence (Table 1). This method of dividing the data ensured that (i) all baseline brain age models were tested on a dataset of the same size using the same examinations and (ii) there was no ‘data leakage’ (i.e. patients in the training set did not appear in the validation or testing sets).

**TABLE 1:** Radiologically normal for age ‘internal clinical datasets’ used for training and testing our baseline brain age models.

Dataset	*N* scans	Age, years (mean ± standard deviation)	Age, years range	Unique patients	Male/female
Axial T_2_‐weighted (training)	18,890	43.7 ± 15.4	18–96	14,507	7685/11,205
Axial DWI (training)	15,553	44.1 ± 15.1	18–96	12,402	6114/9439
Coronal T_2_‐FLAIR (training)	12,228	42.4 ± 14.7	18–94	10,113	4774/7454
Sagittal T_1_‐weighted (training)	7263	42.8 ± 14.4	18–94	6485	2515/4748
Axial GRE T_2_*‐weighted (training)	7306	45.4 ± 15.7	18–94	6295	3141/4165
Validation (all sequences)	1500	42.9 ± 14.7	18–94	1500	574/926
Testing (all sequences)	2000	43.1 ± 14.3	18–94	2000	744/1256

*Note*: The datasets are clinically representative, containing patients of diverse ethnicity (40% non‐white) covering the full adult lifespan (18–96 years).

Henceforth, we refer to these datasets as the ‘*internal clinical datasets*’, to distinguish them from the ‘*out‐of‐sample testing datasets*’ presented in Section 2.1.2.

#### 2.1.2 External ‘out‐of‐sample testing’ datasets

To determine whether there is an improvement in baseline model generalisability following fine‐tuning with transfer learning, images from three publicly accessible datasets were utilised. These datasets included equivalent MRI sequences present in the internal clinical datasets used for baseline brain age model development (Section 2.1.1), along with MRI sequences not typically acquired during routine clinical examinations.

**FIGURE 2:**
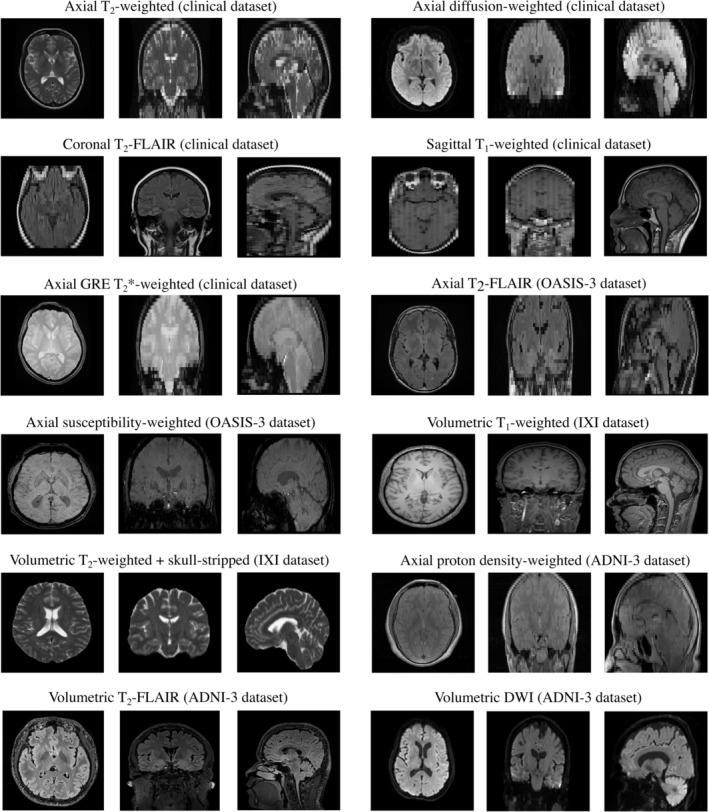
Overview of the different types of head MRI scans used for brain age modelling in this study. A transfer learning experiment using skull‐stripped data is described in Section 2.2.

All axial T_2_‐weighted (*N* = 560), axial DWI (*N* = 389), and volumetric T_1_‐weighted (*N* = 563) scans from the Information eXtraction from Images (IXI) healthy subject dataset were obtained (Table 2). These scans were acquired at three different UK institutions between 2005 and 2008 (Hammersmith Hospital, using a Phillips 3 T system; Guy's Hospital, using a Phillips 1.5 T system; and the Institute of Psychiatry, Psychology and Neuroscience, using a GE 1.5 T system) and can be downloaded from https://brain-development.org/ixi-dataset/. Similarly, all axial susceptibility‐weighted images (SWI) (*N* = 453) and axial T_2_‐FLAIR images (*N* = 381) for the subset of first‐visit, cognitively normal participants from the Open Access Series of Imaging Studies (OASIS‐3) dataset were obtained. These scans were acquired at the Washington University Knight Alzheimer Disease Research Center using three different Siemens scanners (Vision 1.5 T, TIM Trio 3 T, and BioGraph mMR PET‐MR 3 T) and can be downloaded from https://www.oasis-brains.org/. Finally, all axial proton density (PD)‐weighted (*N* = 773), volumetric DWI (*N* = 101), and volumetric T2‐FLAIR (*N* = 503) images for the subset of normal participants only from the Alzheimer's Disease Neuroimaging Initiative (ADNI‐3) dataset were obtained. The scans were performed across 49 sites in the United States (see https://adni.loni.usc.edu/about/centers-cores/study-sites/), using 1.5 T Siemens, 1.5 T GE, and 1.5 T Philips scanners and can be downloaded from https://adni.loni.usc.edu/data-samples/access-data/. Figure 2 provides an overview of all the different types of head MRI scans used in this study.

**TABLE 2:** External, publicly accessible ‘out‐of‐sample testing datasets’ utilised for transfer learning experiments.

MRI sequence	Dataset	*N* scans	Age, years (mean ± standard deviation)	Age range, years	Male/female
Axial T_2_‐weighted	IXI	560	48.6 ± 16.5	20–86	247/313
Volumetric T_1_‐weighted	IXI	563	48.7 ± 16.5	20–86	250/313
Axial DWI	IXI	389	52.3 ± 15.8	20–86	175/214
Axial SWI	OASIS‐3	453	66.0 ± 8.8	42–95	175/278
Axial T_2_‐FLAIR	OASIS‐3	381	68.2 ± 9.1	42–92	156/225
Axial PD‐weighted	ADNI‐3	773	77.2 ± 5.3	61–93	368/405
Volumetric T_2_‐FLAIR	ADNI‐3	503	71.9 ± 8.3	51–95	199/304
Volumetric DWI	ADNI‐3	101	74.2 ± 7.5	55–90	41/60

*Note*: These scans were also used to generate a test set of skull‐stripped, spatially normalised images (Section 2.2).

### 2.2 Neuroimaging processing

We performed minimal pre‐processing of raw head MRI scans. Specifically, axial T_2_‐weighted, axial DWI, coronal T_2_‐FLAIR, sagittal T_1_‐weighted, axial GRE T_2_*‐weighted, axial SWI, axial PD‐weighted, and volumetric T_1_‐weighted images with arbitrary resolution and dimensions, stored as Digital Imaging and Communications in Medicine (DICOM) files, were converted into NIfTI format, resampled to common voxel sizes and dimensions (1.4 mm^3^), and then cropped or padded to achieve a uniform image size (182 mm × 182 mm × 182 mm, corresponding to a 3D array, or ‘tensor’, with dimensions 130 × 130 × 130). Each image's intensity was normalised by subtracting the mean and dividing by the standard deviation. Spatial registration, bias field correction, and skull‐stripping were not performed. All pre‐processing was carried out using open‐source software: dcm2niix (Li et al., 2016) for DICOM‐to‐NIfTI conversion, NiBabel (Brett et al., 2020) for loading and manipulating NIfTI files, and Project MONAI (Cardoso et al., 2022) for resampling and resizing images.

To explore the application of transfer learning to allow our baseline models to generalise to research datasets which often contain high‐resolution images that have been skull‐stripped and spatially registered, a separate processed dataset was created. We removed non‐brain tissue from all 560 axial T_2_‐weighted scans in the IXI dataset using HD‐BET (Isensee et al., 2019), a publicly available deep learning‐based skull‐stripping tool accessible at https://github.com/MIC-DKFZ/HD-BET. The images were then resampled to a uniform voxel size (1 mm^3^) and aligned, via non‐linear registration, to the MNI152 template using ANTsPy (Tustison et al., 2021). The final images measured 182 mm × 218 mm × 182 mm; the corresponding tensors had dimensions 182 × 218 × 182.

### 2.3 Brain age modelling

**FIGURE 3:**
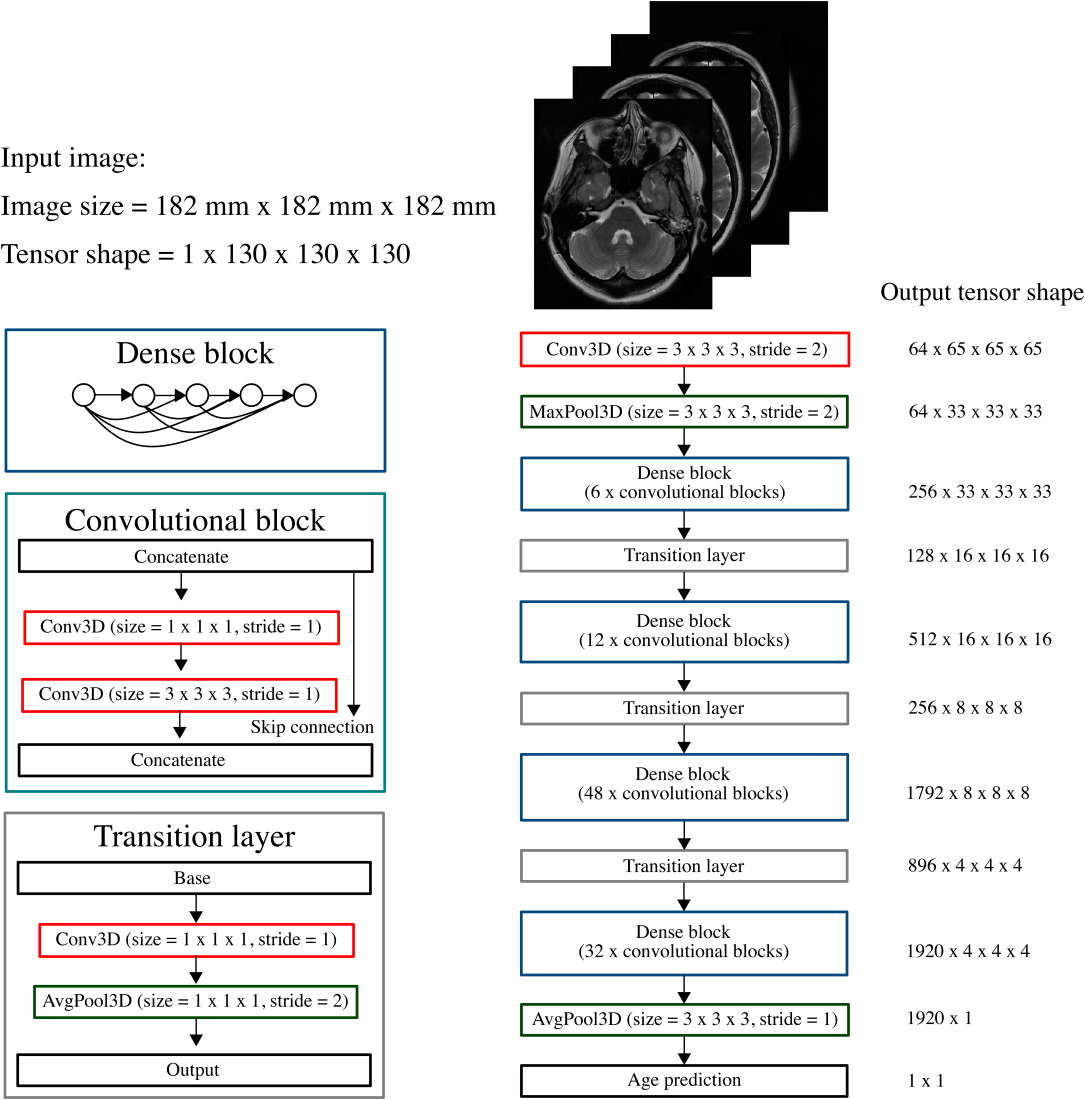
DenseNet201 3D convolutional neural network architecture used in this study. Also shown are the output sizes at each internal layer of the network for an input image of size 182 mm × 182 mm × 182 mm, corresponding to an image tensor of shape 130 × 130 × 130.

Each baseline brain age model was based on the ‘DenseNet201’ architecture (Huang et al., 2017), with modifications to accommodate 3D neuroimaging data. Our network (Figure 3) consists of an initial block of 64 convolutional filters and a ‘max pooling’ layer, followed by four ‘densely connected’ convolutional blocks. Each dense block comprises alternating pointwise and volumetric convolutions which are repeated 6, 12, 48, and 32 times across the four blocks, respectively. Between each dense block are ‘transition layers’ which consist of a point convolution and an average pooling layer. Global average pooling is applied to the output of the final dense block, resulting in a 1920‐dimensional feature vector which is converted by a fully connected layer into a prediction for the patient's age.

We elected to use a standard, pre‐existing network, rather than design a custom architecture, to ensure reproducibility and transparency of our framework. The incorporation of a global average pooling layer in DenseNet201 made it an attractive choice as it allows for the handling of images of different sizes to those encountered during training.

The brain age models used in this study were adapted from the Project MONAI DenseNet201 implementation. All experiments were conducted using PyTorch 1.7.1 (Paszke et al., 2019) with two NVIDIA RTX 2080 graphics processing units (GPUs). Each baseline model was trained by minimising the L1 loss (i.e. absolute error loss) between chronological age and predicted age, with the Adam optimiser (Kingma & Ba, 2014) used to update CNN weights. The batch size was set to 14 as this was the maximum possible size using two 12‐GB GPUs. The learning rate for baseline model training was initially set to 10^−4^ and then reduced by a factor of 2 after every five epochs without improvement on the validation set. In total, each model was trained for 100 epochs; however, checkpoints were saved after each epoch, and the model configuration with the lowest validation set loss was used for testing. In other words, early stopping was employed. The mean absolute error (MAE), Pearson's correlation (*r*), and the coefficient of determination (*R* ^2^) were used to quantify baseline model performance in the internal clinical test sets. Pearson's correlation was also used to quantify the pair‐wise agreement between brain age predictions produced by separate baseline models for patients in the internal clinical test sets. Paired Student's *t* tests were used to test the statistical significance of differences in performance between the baseline models.

We also explored the use of ensemble methods to enhance the accuracy of brain age prediction in our internal clinical test sets. Two different aggregation strategies were applied to combine the predictions of individual baseline models into a single examination‐level prediction of brain age. The first strategy involved a simple mean aggregation approach, whereby the predicted age was obtained by averaging the predictions from each baseline model (i.e. predicted age = [axial T_2_‐weighted prediction + coronal T_2_‐FLAIR prediction + sagittal T_1_‐weighted prediction + axial DWI prediction + axial GRE T_2_*‐weighted prediction]/5). The second strategy utilised a weighted aggregation approach, whereby the predicted age was determined by combining the predictions of each baseline model using different weights (i.e. predicted age = *α*_T2_ * axial T_2_‐weighted prediction + *α*_FLAIR_ * coronal T_2_‐FLAIR prediction + *α*_T1_ * sagittal T_1_‐weighted prediction + *α*_DWI_ * axial DWI prediction + *α*_GRE_ * axial GRE T_2_*‐weighted prediction). We determined the optimal weights (i.e. *α*_T2_, *α*_FLAIR_, *α*_T1_, *α*_DWI_, and *α*_GRE_) by fitting a 5‐parameter linear regression model with no intercept term using predictions obtained for the validation set. Regression modelling was performed using scikit‐learn 0.24.0 (Pedregosa et al., 2011), and all hyperparameters were set to the default values.

We evaluated the performance of our pre‐trained baseline models with out‐of‐sample test set images in three distinct ways. First, we examined model performance without any additional fine‐tuning, which we refer to as ‘*out‐of‐sample testing without transfer learning*’. Second, we applied transfer learning, using 80% of the out‐of‐sample test set data for fine‐tuning and reserving 20% for testing, a process we term ‘*out‐of‐sample testing with transfer learning*’. Finally, we compared these results with those obtained from architecturally identical models that were trained entirely from scratch using the external, publicly available datasets exclusively (i.e. without any transfer learning); we refer to this as ‘de novo *out‐of‐sample training without transfer learning*’. Confidence intervals for the transfer learning and de novo testing approaches were generated using a fivefold cross‐validation procedure, ensuring that each image was only tested once. For model fine‐tuning, the initial learning rate was set to 10^−5^ and then reduced by a factor of 2 after every five epochs without improvement on the out‐of‐sample validation set. All other hyperparameters (i.e. optimiser and mini‐batch size) were identical to those used during the original baseline model training.

**FIGURE 4:**
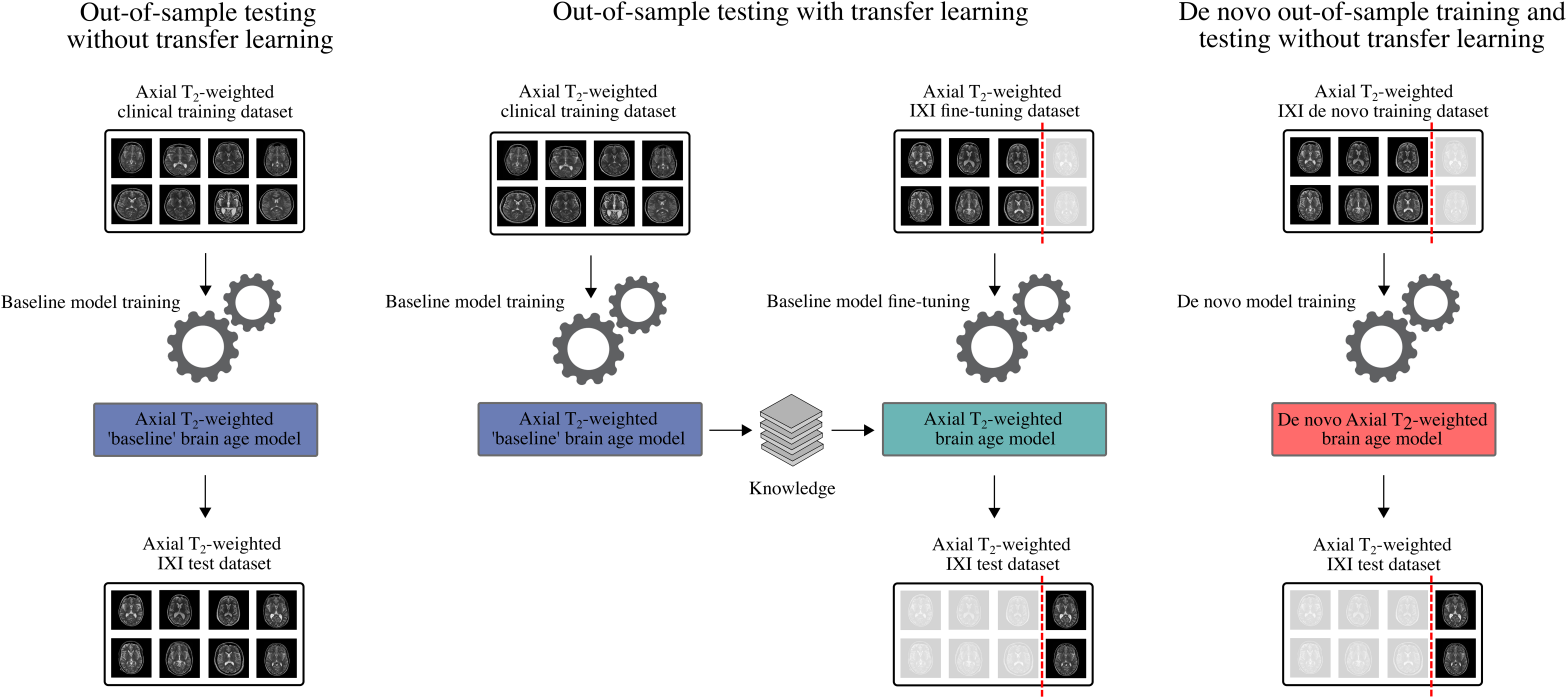
Overview of the three out‐of‐sample testing procedures used in this study. ‘Out‐of‐sample testing without transfer learning’ (left) involves the direct application of baseline models to out‐of‐sample (i.e. IXI, OASIS‐3, or ADNI) data without any further fine‐tuning. ‘Out‐of‐sample testing with transfer learning’ (middle) involves fine‐tuning the baseline models, with a subset of the out‐of‐sample data serving as an additional training set and the remaining out‐of‐sample data used for testing. ‘De novo out‐of‐sample training and testing without transfer learning’ (right) involves training architecturally identical DenseNet201 models from scratch (i.e. without transfer learning), with out‐of‐sample data serving as the training and testing sets.

A summary of our out‐of‐sample testing approaches is provided in Figure 4.

The impact of our transfer learning approach was assessed in three scenarios: (i) using out‐of‐sample MRI sequences and orientations which matched those used to train the corresponding baseline models (e.g. fine‐tuning the axial T_2_‐weighted baseline model with out‐of‐sample axial T_2_‐weighted images); (ii) using closely related sequences and orientations (e.g. fine‐tuning the sagittal T_1_‐weighted model with out‐of‐sample volumetric T_1_‐weighted images); and (iii) using markedly different sequences and orientations (e.g. fine‐tuning the axial T_2_‐weighted model with out‐of‐sample axial susceptibility‐weighted, PD‐weighted images, or even skull‐stripped axial T_2_‐weighted images). An overview of these three transfer learning scenarios is provided in Figure B1 in Appendix B.

To explore the influence of sample size on the baseline model fine‐tuning process, we conducted sample size control experiments. We separately applied transfer learning to baseline models with varying numbers of out‐of‐sample scans serving as the fine‐tuning training dataset (specifically 10, 25, 50, 100, 150, 200, 250, 300, and 350 scans). For each sample size, we used a consistent test set (*N* = 135) and generated confidence intervals by repeating the training and testing process using five separate training datasets randomly sampled from the remaining scans.

Scripts to enable readers to run and fine‐tune our trained baseline models using their own MRI scans are available at https://github.com/MIDIconsortium/BrainAge.

## 3 RESULTS

### 3.1 Brain age prediction using baseline models

**FIGURE 5:**
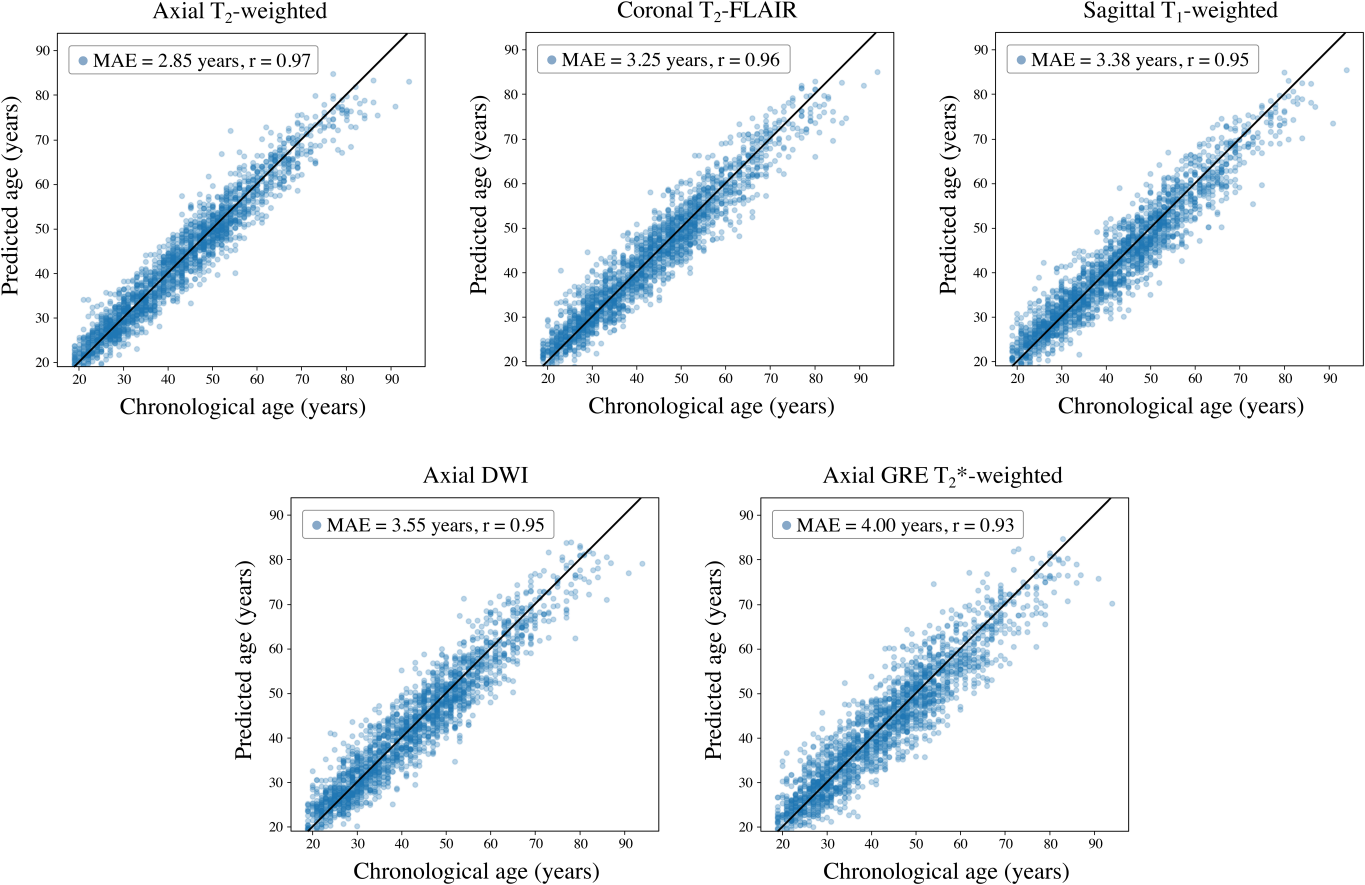
Scatter plots of predicted age versus chronological age in the internal clinical testing sets for each baseline model. Accurate age estimation was achieved for all five models (MAE ≤ 4.0 years, *r* ≥ .93), with the highest accuracy observed using the axial T_2_‐weighted model (MAE = 2.85 years, *r* = =.97).

All baseline models, representing the five commonest sequences and orientations in study dataset, predicted chronological age with high accuracy in the internal clinical testing datasets (MAE ≤ 4.0 years, Pearson's correlation, *r* ≥ .93). The axial T_2_‐weighted model achieved the best test set performance (MAE = 2.85 years, *r* = .97), followed by the coronal T_2_‐FLAIR (MAE = 3.25 years, *r* = .96), sagittal T_1_‐weighted (MAE = 3.38 years, *r* = .95), axial DWI (MAE = 3.55 years, *r* = .95), and axial GRE T_2_*‐weighted (MAE = 4.00 years, *r* = .93) models (Figure 5; Table 3).

**TABLE 3:** Brain age prediction results for the five baseline models considered in this study using the internal clinical testing datasets.

Baseline model	MAE (years)	Pearson's correlation (*r*)	Coefficient of determination (*R* ^2^)
Axial T_2_‐weighted	2.85	.97	.94
Coronal T_2_‐FLAIR	3.25	.96	.92
Sagittal T_1_‐weighted	3.38	.95	.90
Axial DWI	3.55	.95	.90
Axial GRE T_2_*‐weighted	4.00	.93	.86

**FIGURE 6:**
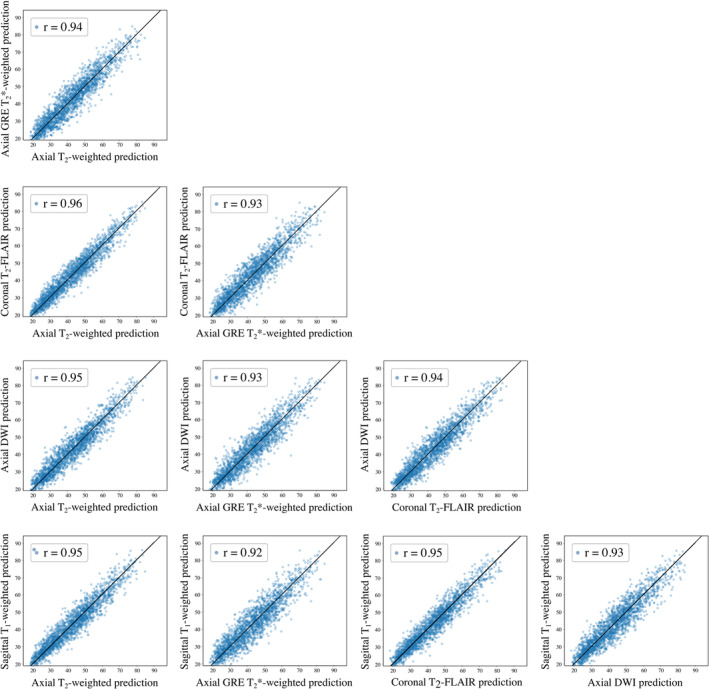
Pair‐wise scatter plots comparing brain age predictions for patients in the internal clinical testing sets using different baseline models. Strong correlation (*r* ≥ .92) between predictions was seen for all pairs of models. To avoid redundancy, no duplicate graphs are shown (the underlying correlation matrix is symmetric with unit diagonal as shown in Figure C1 in Appendix C).

When comparing the five different baseline models with one another, highly correlated pairwise predictions were seen (*r* ≥ .92) (Figure 6).

### 3.2 Ensemble models for enhanced brain age prediction

**FIGURE 7:**
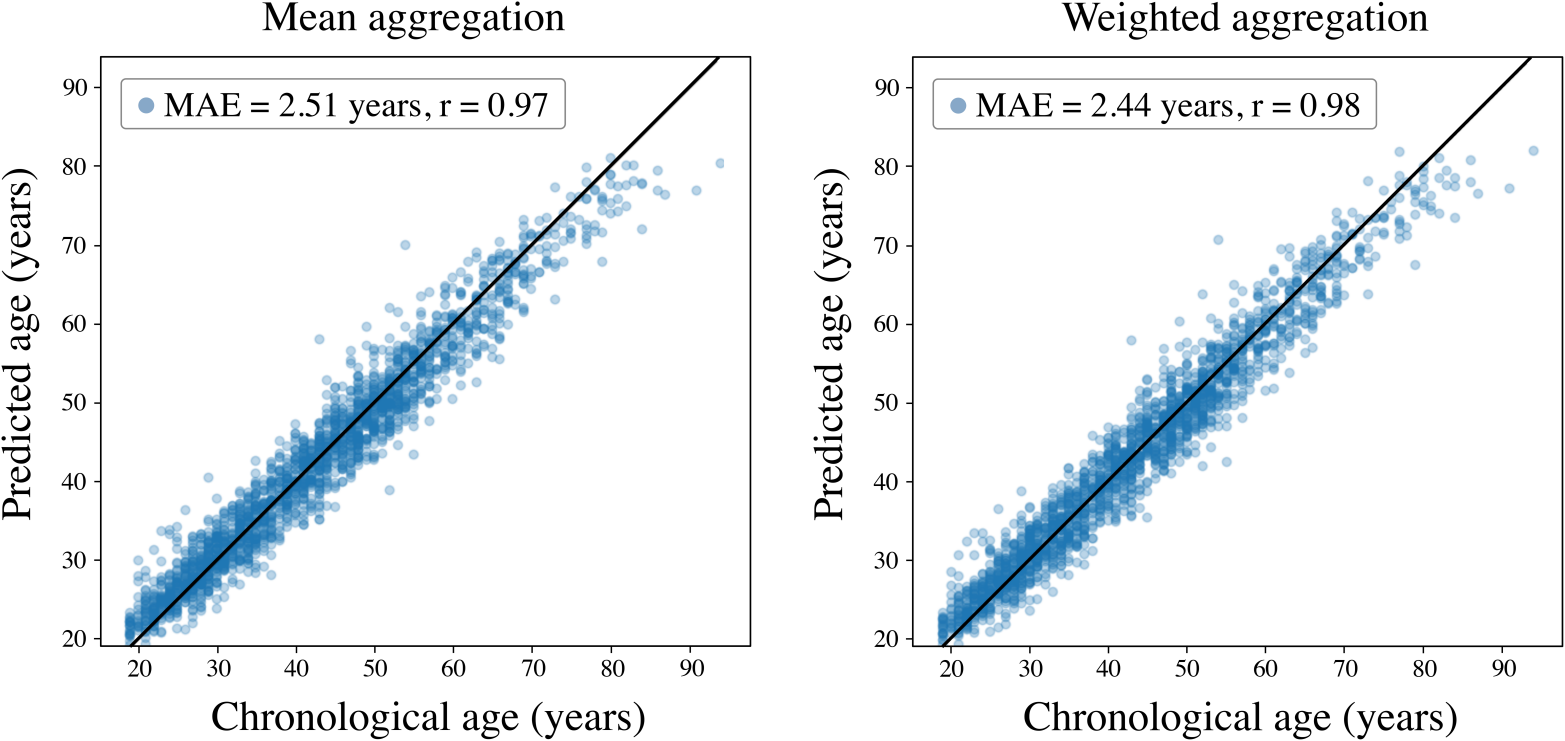
Brain age estimation using ensemble models. Employing mean aggregation to merge individual baseline model predictions resulted in more accurate estimates of brain age compared with using individual baseline models alone (left) (MAE = 2.51 years, *r* = .97). A further improvement in accuracy was achieved by adopting a weighted aggregation approach (right) (MAE = 2.44 years, *r* = .98), with the optimal weights found to be *α*_T2_ = 0.47, *α*_T1_ = 0.17, *α*_DWI_ = 0.17, *α*_FLAIR_ = 0.11, and *α*_GRE_ = 0.09.

An ensemble model which combined the predictions of each baseline model through a simple mean aggregation strategy outperformed all baseline models individually using the same internal clinical testing dataset (*p* < .0001) (MAE = 2.51 years, *r* = .97) (Figure 7; Table 4). Prediction accuracy was further improved using a weighted aggregation strategy (*p* = .00013) (MAE = 2.44 years, *r* = .98). The optimal weights were found to be *α*_T2_ = 0.47, *α*_T1_ = 0.17, *α*_DWI_ = 0.17, *α*_FLAIR_ = 0.11, and *α*_GRE_ = 0.09.

**TABLE 4:** Brain age prediction results for the two ensemble strategies considered in this study: mean aggregation and weighted aggregation.

Ensemble model	MAE (years)	Pearson's correlation (*r*)	Coefficient of determination (*R* ^2^)
Mean aggregation	2.51	.97	.95
Weighted aggregation	3.44	.98	.96

### 3.3 Generalisability to out‐of‐sample testing data

**FIGURE 8:**
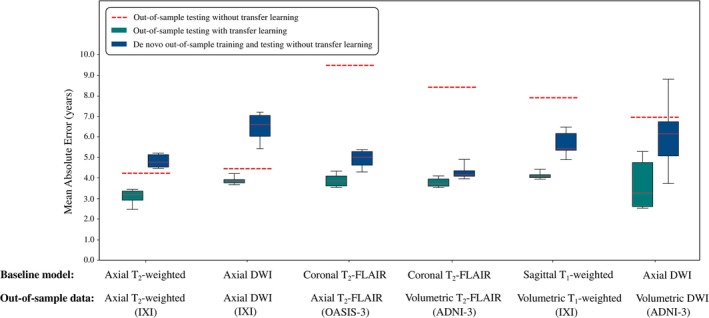
Boxplots showing baseline model generalisability for out‐of‐sample scans. Models were tested (i) without additional training, which we refer to as ‘out‐of‐sample testing without transfer learning’ (dotted red lines), and (ii) after applying transfer learning with a subset of the out‐of‐sample data serving as an additional fine‐tuning training dataset, which we refer to as ‘out‐of‐sample testing with transfer learning’ (green boxes). Comparison was made with additional, architecturally identical models trained ‘from scratch’ (i.e. without transfer learning) using out‐of‐sample data exclusively, which we refer to as ‘de novo out‐of‐sample training without transfer learning’ (blue boxes). In all cases, applying transfer learning outperformed de novo out‐of‐sample training.

Baseline models, tested on out‐of‐sample images with the equivalent sequences and orientations as the corresponding internal clinical training datasets, accurately predicted chronological age without any additional fine‐tuning. In other words, strong out‐of‐sample generalisability was observed. The axial T_2_‐weighted model accurately predicted chronological age using axial T_2_‐weighted scans from the IXI dataset (MAE = 4.21 years, *r* = .96); likewise, the axial DWI model accurately predicted chronological age using axial DWI images, also from IXI (MAE = 4.43 years, *r* = .94) (Figure 8; Figure D1 in Appendix D). Applying transfer learning to these models resulted in improved brain age prediction in the IXI external dataset (axial T_2_‐weighted: MAE = 3.08 years, *r* = .97, *p* < .0001; axial DWI: MAE = 3.87 years, *r* = .95, *p* < .0001). For both IXI MRI sequences, baseline models with and without transfer learning outperformed de novo out‐of‐sample training using architecturally identical models (axial T_2_‐weighted: MAE = 4.83 years, *r* = .93, *p* < .0001; axial DWI: MAE = 9.05 years, *r* = .75, *p* < .0001) (Table 5).

**TABLE 5:** Generalisability of our baseline models to out‐of‐sample images with sequences and orientations that were equivalent or closely related to the corresponding internal clinical training datasets.

Baseline model	Out‐of‐sample test scans	Testing without transfer learning		Testing with transfer learning		De novo out‐of‐sample training	
		MAE (years)	Pearson's correlation (*r*)	MAE (years)	Pearson's correlation (*r*)	MAE (years)	Pearson's correlation (*r*)
Axial T_2_‐weighted	Axial T_2_‐weighted (IXI)	4.21	.96	3.08	.97	4.83	.93
Axial DWI	Axial DWI (IXI)	4.43	.94	3.87	.95	9.05	.75
	Volumetric DWI (ADNI‐3)	6.94	.73	3.69	.79	6.20	.42
Coronal T_2_‐FLAIR	Axial T2‐FLAIR (OASIS‐3)	9.87	.79	3.88	.86	4.80	.76
	Volumetric T2‐FLAIR (ADNI‐3)	8.49	.76	3.78	.83	4.34	.76
Sagittal T_1_‐weighted	Volumetric T_1_‐weighted (IXI)	7.86	.84	4.12	.95	5.66	.90

Baseline models tested on out‐of‐sample images with sequences and orientations that were closely related to the corresponding internal clinical training datasets demonstrated a reduction in generalisability when there had been no additional fine‐tuning. The sagittal T_1_‐weighted model predicted chronological age using volumetric T1‐weighted scans from the IXI dataset with moderate accuracy (MAE = 7.86 years, *r* = .84). The coronal T_2_‐FLAIR model predicted chronological age using axial T_2_‐FLAIR scans from the OASIS‐3 dataset with moderate accuracy (MAE = 9.87 years, *r* = .79); likewise, the coronal T_2_‐FLAIR model predicted chronological age using volumetric T_2_‐FLAIR scans from the ADNI‐3 dataset with moderate accuracy (MAE = 8.49 years, *r* = .76). Finally, the axial DWI model predicted chronological age using volumetric DWI scans from the ADNI‐3 dataset with moderate accuracy (MAE = 6.94 years, *r* = .73). Applying transfer learning with these models resulted in substantial improvements (volumetric T_1_‐weighted: MAE = 4.12 years, *r* = .95, *p* < .0001; axial T_2_‐FLAIR: MAE = 3.88 years, *r* = .86, *p* < .0001; volumetric T_2_‐FLAIR: MAE = 3.78 years, *r* = 0.83, *p* < .001; volumetric DWI: MAE = 3.69 years, *r* = .79, *p* < .001). In all cases, transfer learning outperformed de novo out‐of‐sample training and testing using architecturally identical models (volumetric T_1_‐weighted: MAE = 5.66 years, *r* = .90, *p* < .0001; axial T_2_‐FLAIR: MAE = 4.80 years, *r* = .76, *p* < .0001; volumetric T_2_‐FLAIR: MAE = 4.34 years, *r* = .76, *p* < .001; volumetric DWI: MAE = 6.20 years, *r* = .42, *p* < .001) (Figure 8; Figure D2 in Appendix D; Table 5).

**FIGURE 9:**
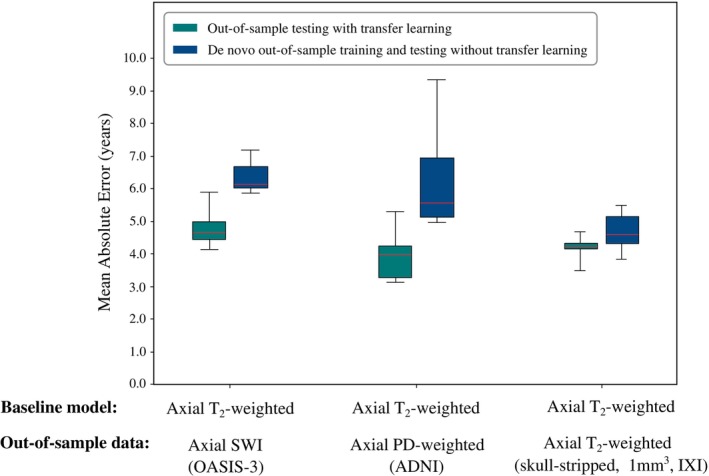
Boxplots showing the impact of transfer learning when applied to images markedly different from those in the corresponding internal clinical datasets used for baseline model training. Here, the axial T_2_‐weighted baseline model was fine‐tuned to predict brain age in out‐of‐sample axial SWI, axial PD‐weighted, and skull‐stripped, spatially normalised axial T_2_‐weighted images. Comparison is made with de novo out‐of‐sample training. In all cases, transfer learning (green boxes) outperformed ‘de novo training without transfer learning’ (blue boxes). Note that the results from ‘out‐of‐sample testing without transfer learning’ are not shown as these are all MAE > 10 years.

Baseline models tested on out‐of‐sample images with sequences that were markedly different to the corresponding internal clinical training datasets demonstrated a large reduction in generalisability when there had been no additional fine‐tuning. The best individual baseline model (axial T_2_‐weighted) failed to predict chronological age accurately (axial SWI scans: MAE = 20.94 years; axial PD‐weighted scans: MAE = 26.54 years; axial T_2_‐weighted scans with 1 mm^3^ voxels, brain tissue removed and spatially registered: MAE = 11.34 years). However, applying transfer learning with this baseline model resulted in substantial improvements (axial SWI scans: MAE = 4.65 years; axial PD‐weighted scans: MAE = 3.92 years; axial T_2_‐weighted scans with 1 mm^3^ voxels, brain tissue removed and spatially registered: MAE = 4.21 years). Again, transfer learning outperformed de novo out‐of‐sample training with architecturally identical models (axial SWI scans: MAE = 6.50 years, *p* < .0001; axial PD‐weighted scans: MAE = 7.78 years, *p* < .0001; axial T_2_‐weighted scans with 1 mm^3^ voxels, brain tissue removed and spatially registered: MAE = 4.51 years, *p* = .023) (Figure 9).

### 3.4 Dataset size control analysis

**FIGURE 10:**
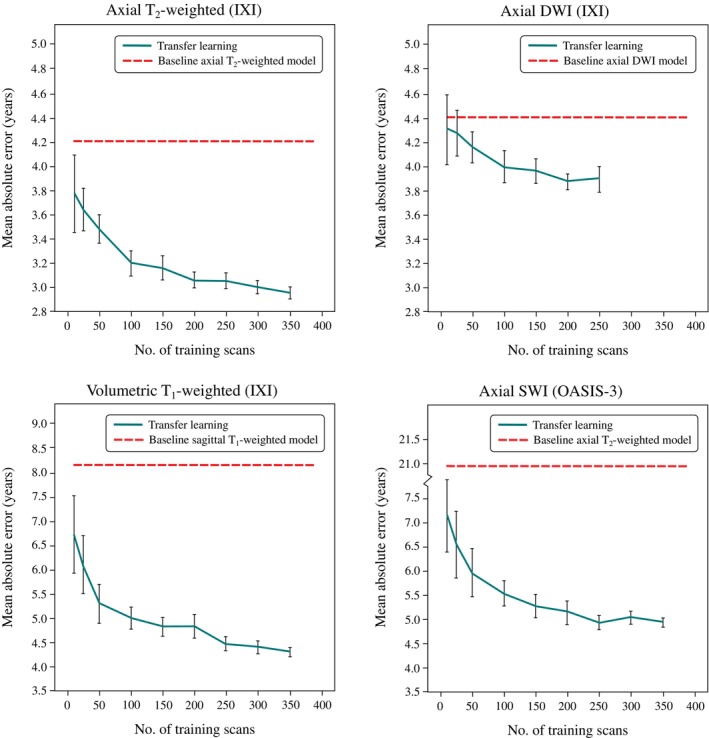
Transfer learning dataset size control analysis. Shown is the testing set MAE as a function of out‐of‐sample fine‐tuning training dataset size for axial T_2_‐weighted (top left), axial DWI (top right), volumetric T_1_‐weighted (bottom left), and axial SWI images from the IXI and OASIS‐3 datasets, using the axial T_2_‐weighted, axial DWI, sagittal T_1_‐weighted, and axial T_2_‐weighted baseline models, respectively. In all cases, rapid improvement (i.e. decreased MAE) was observed using as few as 25–100 scans, with improvements plateauing using fine‐tuning training datasets larger than 200 scans.

By applying transfer learning using different fine‐tuning training sample sizes, we observed that substantial improvements in age estimation can be achieved with only modest quantities of out‐of‐sample scans. In all three scenarios (i.e. when applied to scans matching, or similar to, or markedly different from those in the corresponding internal clinical training datasets), baseline model performance rapidly improved with as little as 25–100 out‐of‐sample scans and plateaued with dataset sizes greater than 200 (Figure 10).

## 4 DISCUSSION

In this study, we have presented an accurate, robust, and generalisable deep learning framework for brain age prediction using a variety of common MRI sequences. Our results emphasise the value of training at scale using large and diverse training datasets and underscore the importance of ensemble methods and transfer learning in improving accuracy and generalisability.

Several key elements distinguish our study. First, the use of a cutting‐edge, transformer‐based neuroradiology report classifier enabled us to generate a large, clinically representative training dataset (Wood et al., 2020, 2021, 2022). This step successfully overcame a significant obstacle often faced in brain age model development (i.e. identifying radiological normal scans in a large hospital dataset), resulting in a diverse and realistic set of training data that accurately represents clinical populations (Agarwal et al., 2023; Booth et al., 2023; Din et al., 2023). The diversity of our data, encompassing a range of scanner vendors, acquisition protocols, patient ethnicities, and a wide age span (18–96 years), added robustness to our models. As a result, our baseline models demonstrated strong generalisation with out‐of‐sample data and formed an effective basis for further enhancements through ensemble methods and transfer learning.

Ensemble methods are another key component of our study. These methods integrate the strengths of multiple distinct models, thereby enhancing prediction performance and reducing individual model biases. In the context of multi‐sequence brain age prediction, ensemble methods offer a unique advantage. They allow predictions from models trained on diverse MRI sequences to be combined, effectively harnessing complementary information from different sequences that may capture different aspects of brain ageing. In our study, we implemented two ensemble aggregation strategies: mean and weighted. Both strategies outperformed all individual baseline models, clearly demonstrating the value of information integration. Notably, our weighted aggregation strategy indicated that different MRI sequences contribute to prediction accuracy to varying degrees. This insight, which may reflect the differential sensitivity of various sequences to certain aspects of brain ageing, could inform future research by guiding the selection of the most informative sequences or those that provide complementary information (Cole et al., 2020; Wood et al., 2019).

The successful application of transfer learning was another key aspect of our study. Transfer learning, the technique of using knowledge gained from one task to improve performance on a related but different task, has been shown to be well suited for brain age prediction (Chen et al., 2020; Jonsson et al., 2019; Leonardsen et al., 2022). The inherent diversity of MRI data (either within the clinic or research settings), such as differences in resolution, field strength, sequence weighting, and orientations, can make it challenging to develop generalisable models.

Unlike much existing work that predominantly relies on models pre‐trained on unrelated tasks, such as ImageNet for image recognition (Bashyam et al., 2020; Jiang et al., 2019; Lin et al., 2021), our study uniquely capitalises on pre‐trained brain age models for fine‐tuning. By doing so, we highlight the potential benefits of domain‐specific pre‐training, which likely provides an advantage in model adaptation with knowledge directly relevant to the current task. This approach allows our models to leverage domain‐specific features and patterns, improving their performance on brain age prediction.

In our study, transfer learning played two key roles. It improved the generalisability of our models to out‐of‐sample scans that closely matched the training data, and importantly, it enabled model adaptation for use with scans underrepresented in the training data. Furthermore, our dataset size control analysis indicated that effective fine‐tuning could be achieved with a very small number of scans, in some cases as low as *N* = 25, thus supporting the potential utility of transfer learning for brain age prediction, even in settings with very limited data.

The ability to fine‐tune our models to fit various clinical scenarios and MRI sequences suggests a potential for wider application of brain age estimation. Such fine‐tuned models could be used across a broad range of neurological and psychiatric disorders, as well as in healthcare settings with diverse MRI technologies and practices. Furthermore, the fact that this fine‐tuning can be accomplished with limited datasets indicates the potential for extending brain age estimation to a variety of patient groups, potentially with varying demographics and varying MRI sequences.

Our study has some limitations. While our models exhibited strong generalisability across different MRI sequences, we did not evaluate their performance on more specialised sequences (e.g. perfusion imaging) or in specific clinical scenarios (e.g. for a given diagnosis). Future investigations should focus on evaluating the performance of the models in these specialised scenarios to validate their applicability. Additionally, the level of improvement gained through transfer learning may vary depending on the degree of similarity between the original and new tasks. Further investigation is needed to understand the extent to which transfer learning can enhance performance in different scenarios. Furthermore, the varying sizes of our baseline model training datasets might have influenced the derived weights in the ensemble model, potentially biasing the contribution of each sequence. Therefore, caution should be exercised when interpreting the weights obtained from the ensemble models, and future studies should explore methods to mitigate this potential bias if it proves to be relevant.

In conclusion, our study presents a flexible and effective approach for brain age prediction using MRI data. By demonstrating the power of ensemble methods and transfer learning, we aim to inspire further exploration in this area and potentially others within the field of neuroradiology. Future studies should address the limitations mentioned and further validate the performance and applicability of the proposed framework in specialised clinical contexts.

By making our pre‐trained models openly accessible, we hope to provide the scientific community with a versatile toolkit that can be used directly or further fine‐tuned to suit the specific requirements of different clinical scenarios and MRI sequences.

## AUTHOR CONTRIBUTIONS

**David A. Wood**: Methodology; software; formal analysis; and writing—original draft preparation. **Matthew Townend**: Software and writing—original draft preparation. **Emily Guilhem**: Data curation and validation. **Sina Kafiabadi**: Data curation and validation. **Ahmed Hammam**: Data curation and validation. **Yiran Wei**: Validation and writing—review and editing. **Ayisha Al Busaidi**: Data curation and validation. **Asif Mazumder**: Data curation and validation. **Peter Sasieni**: Conceptualisation and funding acquisition. **Gareth J. Barker**: Writing—review and editing. **Sebastien Ourselin**: Project administration and funding acquisition. **James H. Cole**: Conceptualisation; supervision; funding acquisition; and writing—review and editing. **Thomas C. Booth**: Conceptualisation; supervision; funding acquisition; and writing—review and editing.

## FUNDING INFORMATION

This work was supported by the Royal College of Radiologists, King's College Hospital Research and Innovation, King's Health Partners Challenge Fund, NVIDIA (through the unrestricted use of a GPU obtained in a competition), the Wellcome/Engineering and Physical Sciences Research Council Center for Medical Engineering (WT 203148/Z/16/Z), and an MRC DPFS grant (MR/W021684/1).

## CONFLICT OF INTEREST STATEMENT

Co‐author Sebastien Ourselin is the co‐founder of Brainminer; however, he did not control or analyse the data. The other authors of this manuscript declare no relationships with any companies whose products or services may be related to the subject matter of the article.

## DATA AND CODE AVAILABILITY

Scripts to enable readers to run our trained brain age models using their own scans are available at https://github.com/MIDIconsortium/BrainAge. The internal clinical datasets used in this study are not publicly available because the IRB of the study limits access to the data. Derived and supporting data are available from the corresponding author upon reasonable request.

## Supporting information


Appendix S1.

## Data Availability

The data that support the findings of this study are available from the corresponding author upon reasonable request.
