# Supplementary material for: Optimising brain age estimation through transfer learning: A suite of pre‐trained foundation models for improved performance and generalisability in a clinical setting
Source: Hum Brain Mapp. 2024 Mar 4;45(4):e26625. doi: 10.1002/hbm.26625 (PMC10910262; doi:10.1002/hbm.26625)
Supplement: Supplementary file 1 — Appendix S1. [file HBM-45-e26625-s001.docx]

**Appendix A**

**
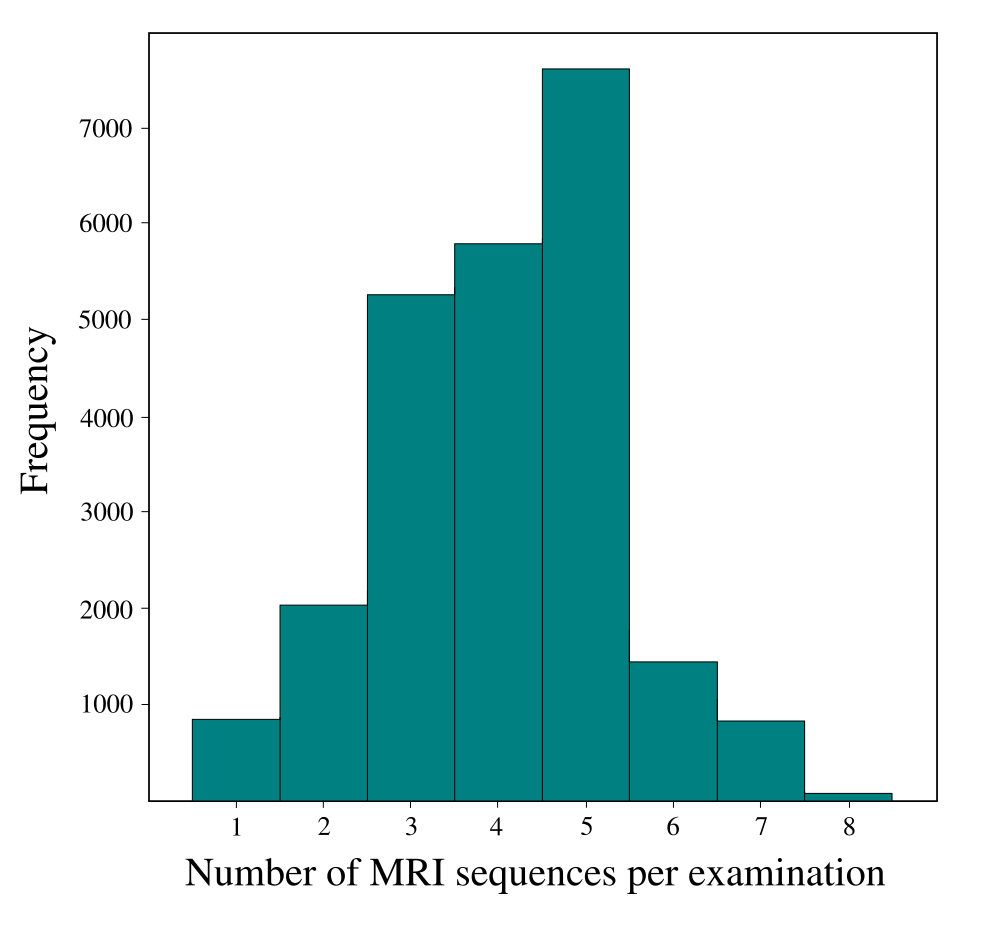
**

**Figure A1:** *Histogram showing the number of MRI sequences acquired per examination at King's College Hospital NHS Foundation Trust (KCH) Guy's and St Thomas’ NHS Foundation Trust (GSTT).*

**Appendix B
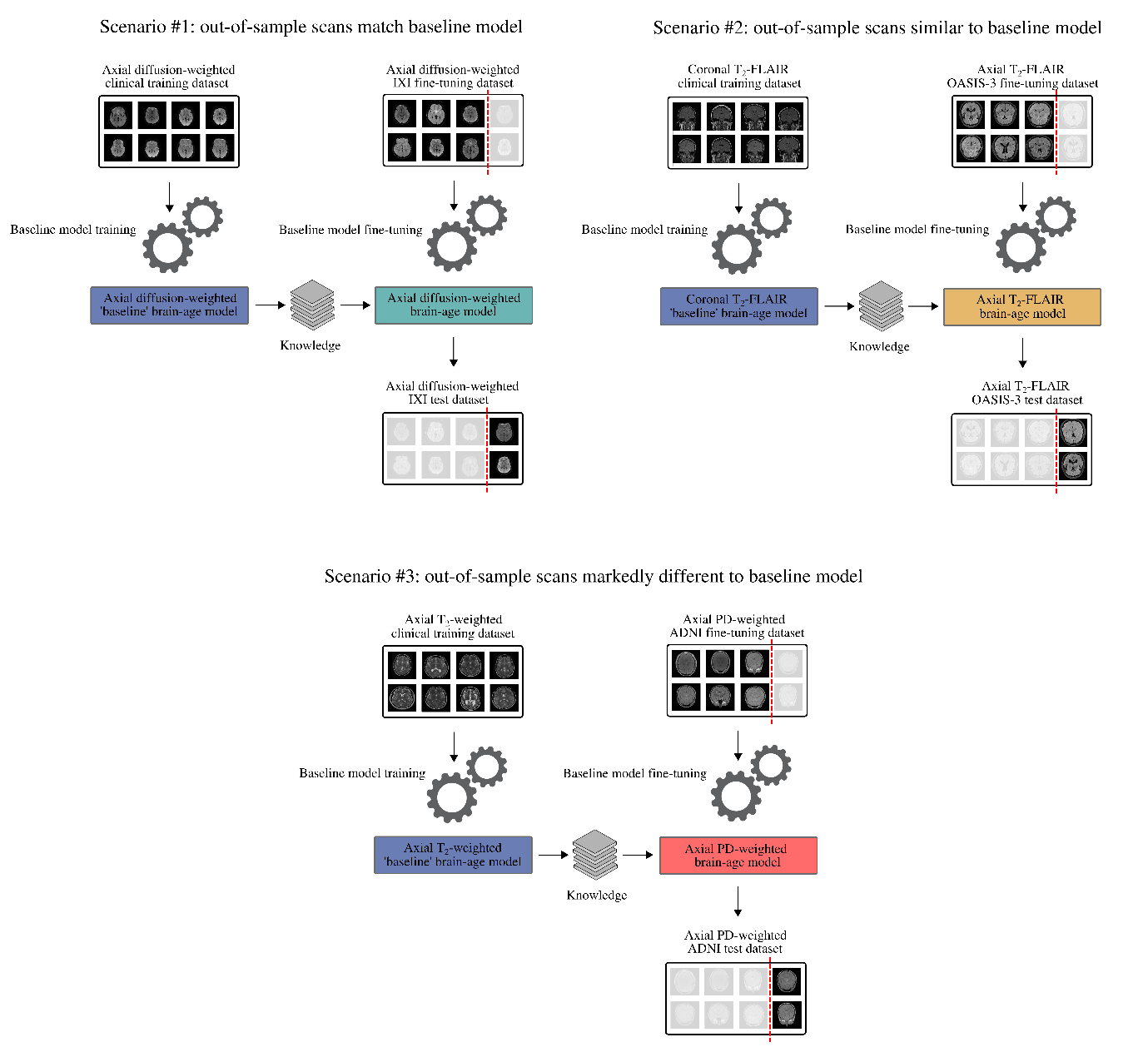
**

**Figure B1:** *Schematic representation of the three scenarios used to evaluate the impact of transfer learning in this study. Scenario #1 (top left) involves fine-tuning a baseline model with out-of-sample MRI sequences and orientations matching those it was trained on (e.g., refining the axial DWI baseline model using out-of-sample axial DWI images). Scenario #2 (top right) involves using closely related, but not identical, sequences and orientations for fine-tuning (e.g., refining the coronal T_2_-FLAIR model using out-of-sample axial T_2_-FLAIR images). Scenario #3 involves fine-tuning the baseline model with markedly different sequences and orientations (e.g., refining the axial T_2_-weighted model with out-of-sample axial PD-weighted images).*

**Appendix C**

**
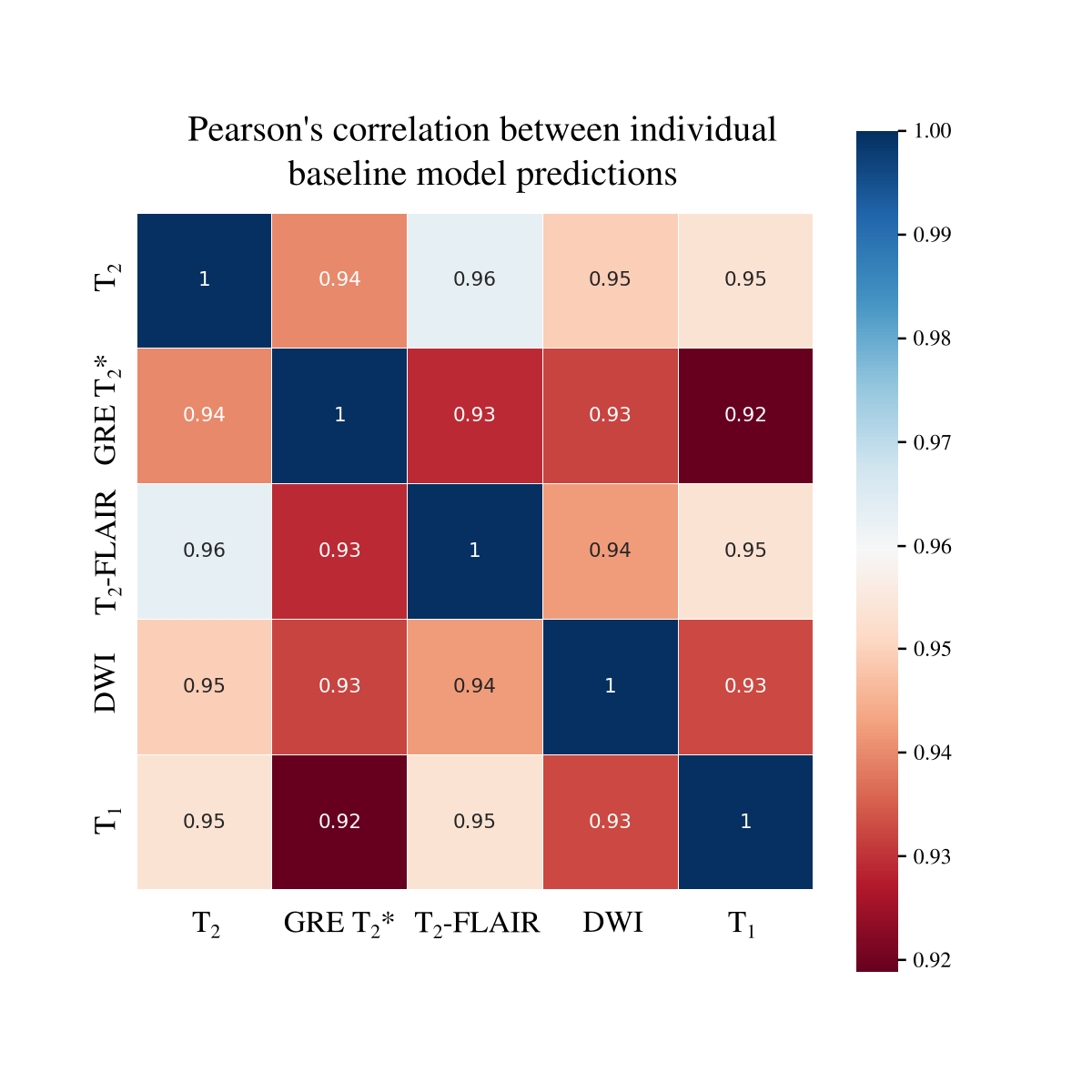
**

**Figure C1:** *Pearson’s correlation matrix, showing the agreement between brain-age predictions for each patient in the testing set using different baseline models. Strong correlation (r ≥ 0.92) was observed for all pairs of MRI sequences.*

**Appendix D**


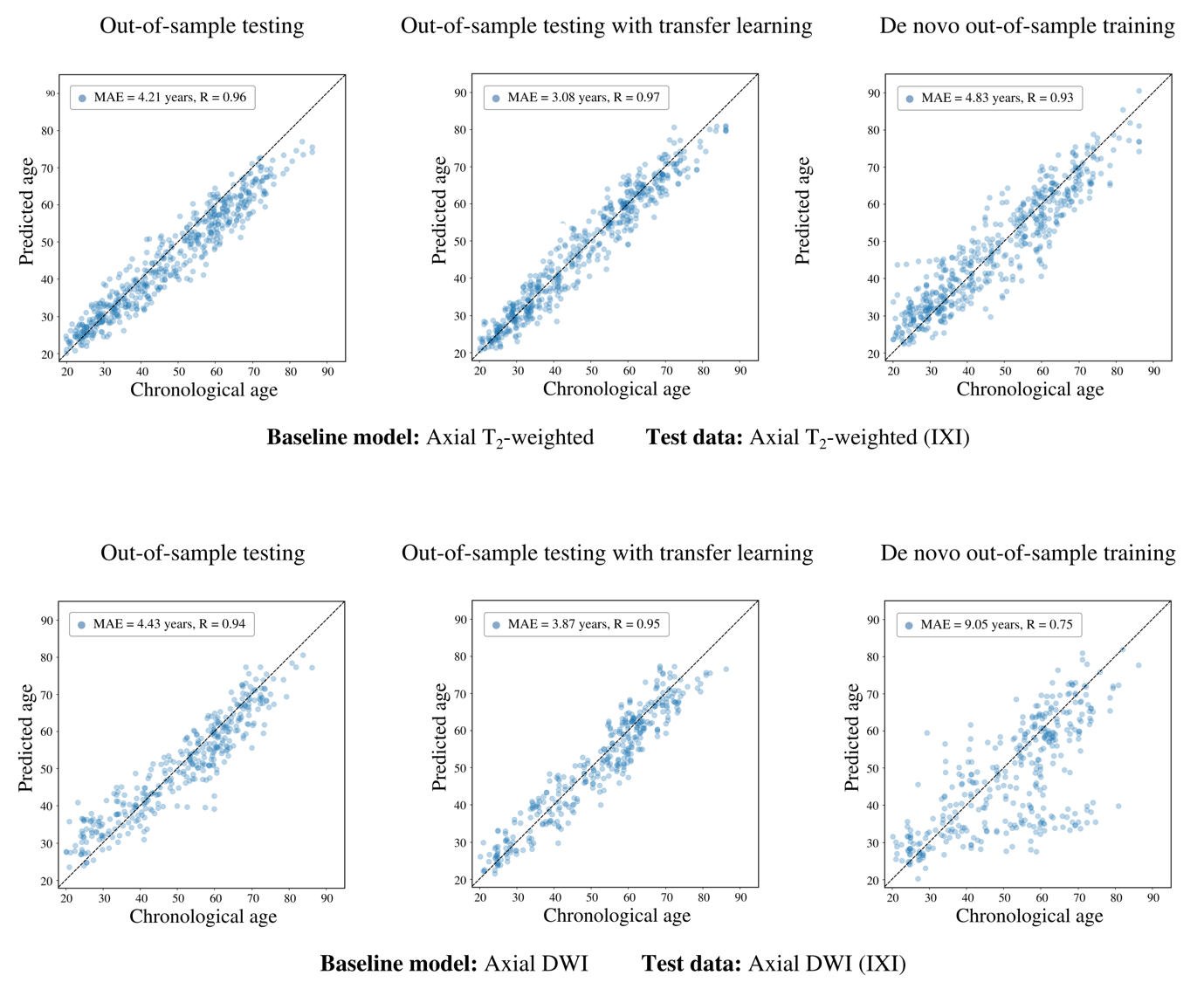
**Figure D1:** *Scatter plots of predicted vs. chronological age for baseline models using out-of-sample scans matching those in the hospital training datasets. Left: accurate brain-age prediction was observed for axial T_2_-weighted* *and axial DWI scans from the external IXI dataset without additional out-of-sample transfer learning (axial T2-weighted: MAE = 4.21 years, r = 0.96; axial DWI: MAE = 4.43 years, r = 0.94). Middle: applying transfer learning to the corresponding baseline models using a subset of the IXI data led to improved out-of-sample prediction accuracy (axial T_2_-weighted: MAE = 3.08 years, r = 0.97; axial DWI: MAE = 3.87 years, r = 0.95). Right: both out-of-sample testing with and without transfer learning outperformed de novo out-of-sample training and testing using architecturally identical models* *and out-of-sample data exclusively (right) (axial T_2_-weighted: MAE = 4.83 years, r = 0.93; axial DWI: MAE = 9.05 years, r = 0.75).*

*
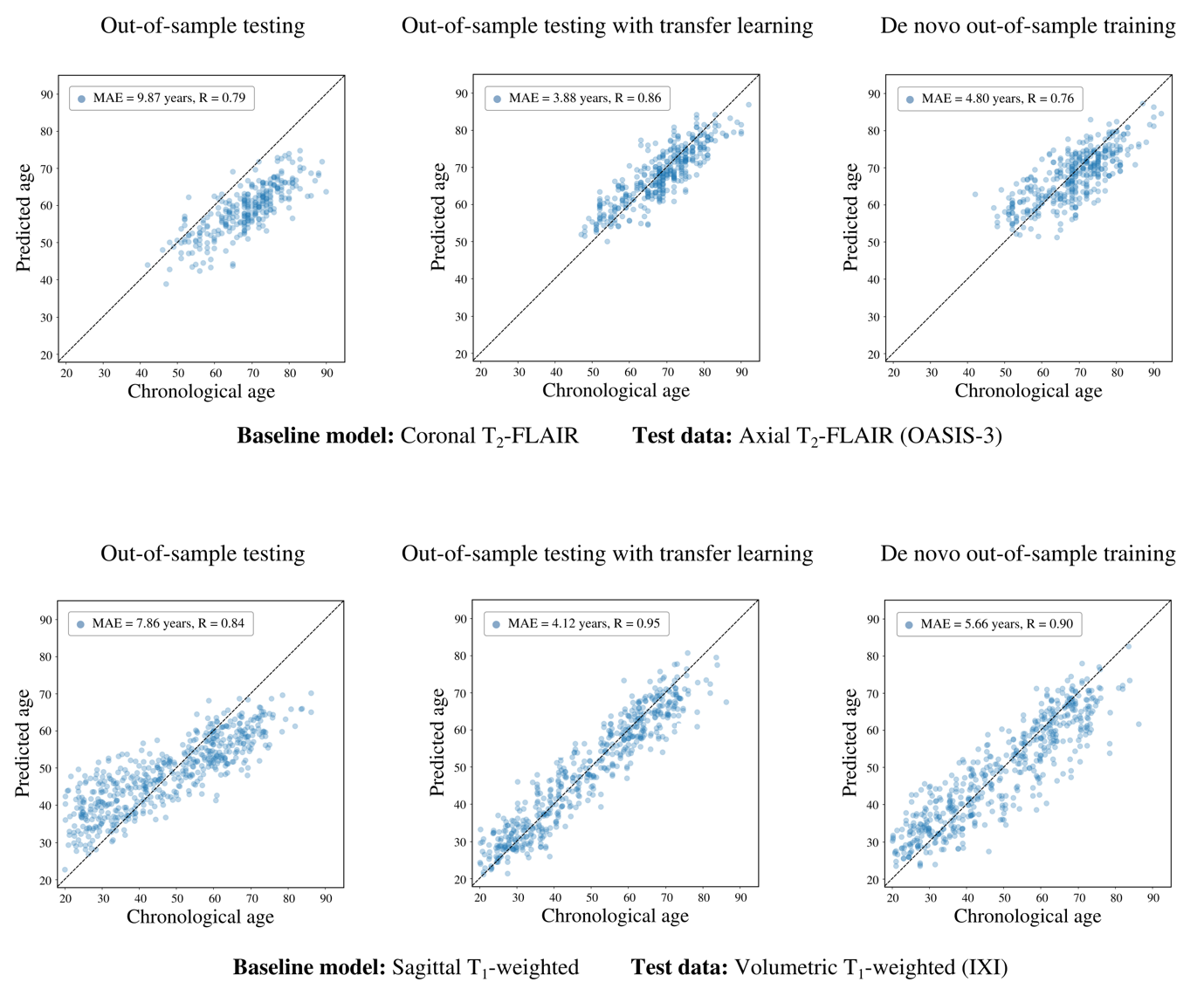
*

**Figure D2:** *Scatter plots of predicted vs. chronological age for baseline models using out-of-sample scans similar, but not identical, to those in the corresponding internal clinical training datasets. Moderate generalisability was observed when applying the coronal T_2_-FLAIR and sagittal T1-weighted baseline models to axial T_2_-FLAIR* *and volumetric T_1_-weighted scans from the external OASIS-3 and IXI datasets, respectively (left) (axial T_2_-FLAIR: MAE = 9.87 years, r = 0.79; volumetric T_1_-weighted: MAE = 7.86 years, r = 0.84). Applying transfer learning with these models resulted in substantial improvements (middle)(volumetric T_1_-weighted: MAE = 4.12 years, r = 0.95; axial T_2_-FLAIR: MAE = 3.88 years, r = 0.75). In both cases, transfer learning outperformed de novo out-of-sample training using architecturally identical models and out-of-sample data exclusively (right) (axial T_2_-FLAIR: MAE = 4.80 years, r = 0.76; volumetric T_1_-weighted: MAE = 5.66 years, r = 0.90).*
